# Transitional Care Experiences of Patients with Hip Fracture Across Different Health Care Settings

**DOI:** 10.5334/ijic.4720

**Published:** 2021-04-08

**Authors:** Laura Brooks, Paul Stolee, Jacobi Elliott, George Heckman

**Affiliations:** 1 University of Waterloo, CA

**Keywords:** care transitions, older adults, integrated care, hip fracture, continuity of care

## Abstract

**Background:**

Transitions of care often result in fragmented care, leading to unmet patient needs and poor satisfaction with care, especially in patients with multiple chronic conditions. This project aimed to understand how experiences of patients with hip fracture, caregivers, and healthcare providers differ across different points of transition.

**Methods:**

A secondary analysis of 103 qualitative, semi-structured interviews was conducted using emergent coding techniques, to gain an understanding of how transitional care experiences may differ across varying settings of care. Following the secondary analysis, a focus group interview was conducted to review findings.

**Results:**

Seven key themes, each relating to distinct transition points, emerged from the secondary analysis: (1) Multiple providers contributed to patient and caregiver confusion; (2) Family caregivers were not considered important in the patient’s care; (3) System-related issues impacted experiences; (4) Patients and caregivers felt uninformed; (5) Transitions increased stress in patients and caregivers; (6) Care was not tailored to patient needs; (7) Providers faced barriers in getting adequate information. The focus group results built upon these themes, adding some additional context to understand the current transitional care landscape.

**Discussion:**

In transitions to formal care settings, similarities were related to feeling confused, while in transitions to home, similarities existed in regards to feeling unprepared. These findings support the view that models of integrated care should consider the context to which they are applied.

## Background

Older adults with multiple chronic conditions typically require care from numerous specialized health care providers, who often practice independently across a variety of different settings [1]. As such, it becomes necessary for patients managing their multiple health concerns to transition between multiple health care providers and a range of settings [2]. These ‘handoffs’ often result in fragmented care, frequently leading to unmet patient needs, adverse events, and poor satisfaction with care, especially in patients with multiple chronic conditions [3,4,5]. The brief and passive visits, practice teams that are lacking information and preparation from previous settings, and under-informed patients which are characteristic of an acute-focused health care system are not conducive to chronic care, and could contribute to rising health care costs [6,7]. A study assessing the patterns of multimorbidity determined that hip fracture was the least likely condition to occur in the absence of a comorbidity [8], making these patients an ideal case for the examination of complex health conditions. Additionally, patients with hip fracture, the majority of whom are older adults with complicated co-morbidities [8,9], tend to face three or more transitions throughout their recovery [10,11]. Transitions of care often require patients to move between different settings. However, different health settings tend to operate in silos, leaving providers under-informed about the problems addressed, services provided, medications prescribed and preferences expressed in previous settings, leaving transitioning patients particularly vulnerable [1,12].

Integrated care is an approach that aims to reduce fragmentation by enhancing coordination and continuity [13]. A number of suggestions for enhancing quality of care transitions have centered on enhancing health care service integration. Specifically, care transitions are thought to be improved through the integration of services within a particular hospital, vertical integration of health services, and horizontal integration of community-based services [14]. Brown and Menec [14] state that existing research on older adults who benefit from integrated care has investigated integration at the level of a single organization or clinic, with very little investigation into integration across health care settings. In the literature, one review of existing care transitions intervention models examined six well-researched models: the Transitional Care Model, Care Transitions Intervention, Project BOOST, Project RED, the Chronic Care Model, and INTERACT [15], concluding that all demonstrated beneficial results in reducing readmissions, hospital utilization, and cost. There is, however, some evidence to suggest that this effectiveness may not be universal. For example, in an evaluation in one hospital, Project RED had no significant impact on readmission or emergency department use [16]. Furthermore, these interventions have largely been developed for patients with particular chronic conditions, such as heart disease, stroke, COPD, diabetes, and mental health conditions [17]. The Transitional Care Model, for example, was created for and tested with patients with heart conditions, which may limit its generalizability to other chronic conditions [18]. Patients with these singular chronic conditions may have a different transitional care journey from patients with hip fracture. Older patients with hip fractures are complex and tend to face more transitions [19]. The effectiveness of these models may be further impacted by the fact that each of these models were designed for implementation in a specific transition setting: from hospital to home of the community setting. Focusing on one transition provides an understanding of a fragment of the patient’s overall experience [20,21], while examining the entire care journey as one event may limit insight into differences that may exist between transitions to different settings. Through an examination of the transitional care journey as a whole, Toscan and colleagues [20] found evidence that experiences are not entirely consistent across different settings of care. Enderlin and colleagues [15] actually express that, because of variations observed between different settings, transitional care interventions should only be used in the context for which they were created.

The care journey of older patients with hip fracture was previously investigated in the InfoRehab Transitions project. In this project, Canadian patients, their family caregivers, and health care providers were interviewed at each transition point to understand their experiences across the entire care journey. This project did not, however, explicitly investigate how experiences at each transition may have differed depending on the specific settings of each transition. Separating the transitions within each patient’s entire care trajectory may provide a more detailed view of each transition while still considering the broader care journey. This may allow for an understanding of how the same patient’s, family caregiver’s and health care provider’s needs, experiences and perspectives differ across settings in their care journey. Understanding how patient, caregiver, and provider needs, experiences, and perspectives differ at various transition points may provide valuable information for cross-setting integration efforts.

This work aimed to answer the following research question: How do the experiences of patients, their family caregivers, and health care providers interviewed in the InfoRehab Transitions project, differ across different transition points? Specifically, this study aims to:

1. To identify experiences and characteristics specific to particular transition points across four specific transitions:1) from acute care to inpatient rehabilitation, 2) from acute care to home, 3) from acute care to long term care, 4) from inpatient rehabilitation to home, through a secondary analysis of patient, family caregiver, and health care provider transcripts.
2. To understand how the emerging themes from the secondary analysis relate to current practice and experiences of health care professionals, and to identify potential gaps in the secondary analysis findings, through a focus group interview.

## Methods

A total of 103 semi-structured interview transcripts from 75 different participants (n = 40 patient interview transcripts, n = 17 informal caregiver interview transcripts n = 46 health care provider interview transcripts) were analyzed by one reviewer using line-by-line emergent coding in the software program NVivo 11. All interviews analyzed for this project were previously conducted in the InfoRehab Transitions study, which followed an ethnographic approach [22]. The interviews that were analyzed occurred across three study sites, in three provinces in Canada: one large urban location, one small urban location, and one rural location. Within both urban locations, researchers partnered with acute care hospitals and rehabilitation hospitals, as well as homecare services. In the rural location, researchers partnered with the hospital which provided rehabilitation services on site, as well as home care services. The interviews occurred at transitions from acute care to home, acute care to long term care, acute care to inpatient rehabilitation and inpatient rehabilitation to home.

The InfoRehab study used a purposeful sampling strategy, described by Patton [23], to recruit patients and their family caregivers post-hip surgery within acute care. Since patients with hip fracture frequently experience vastly different care trajectories, patients were purposefully selected in order to better understand information transfer issues across a variety of representative transitions. A minimum of two health care providers involved in admission or discharge of the patient at each setting were also invited to participate in an interview.

Prior to the secondary analysis, each interview transcript was read in its entirety to ensure the researcher was familiar with the data [24]. After reading, the researcher sorted the transcripts into one of four categories based on the transition in which the interview took place. The four categories were: (1) acute to home, (2) acute to inpatient rehabilitation, (3) acute to long term care, and (4) inpatient rehabilitation to home. There was a minimum of twelve interview transcripts per category. The data was additionally coded according to informant type (patient, caregiver, or health care provider). Data were analyzed using emergent coding techniques according to Lofland, Snow, Anderson & Lofland [25] and Braun & Clarke [24].

The initial coding process was followed by a more structured theming process [24]. This process built upon the initial coding to organize the initial nodes into larger conceptual topics appropriate for further elaboration [24,25]. Prior to the theming process, the final nodes for each patient and family caregiver transcript were labeled by the transition category. The nodes from each distinct transition category were themed separately. The final themes were then matched across transitions to develop an understanding of the overlap of themes across various transitions and settings.

Each of the developed themes was recorded and refined, leading to the development of a final thematic map [24]. The themes were then named and defined to explain the key aspects of each theme [24]. After defining each theme, an analysis of the overlap was completed by comparing and contrasting the themes within each transition category. During this stage, some themes were combined, where possible, to demonstrate the similarities and differences between transitional settings.

In addition to the secondary analysis, a focus group interview with health care providers was conducted. The purpose of the focus group interview was to understand how the results may relate to current practice, and about themes that may be missing from the data. Recruitment for this focus group interview occurred through existing relationships with a regional committee focused on system solutions for older adult issues within health care. During the focus group interview, the researcher presented the results of the secondary analysis and asked for feedback about how the results may relate to current practice, and about themes that may be missing from the data. The focus group interview was audio recorded, transcribed verbatim, and analyzed according to the process outlined above. The emerging themes from the focus group interview were compared with the secondary analysis themes, and reported on accordingly.

After the secondary analysis and the focus group data analysis were completed, emerging themes were examined in relation to existing published research resulting from the InfoRehab project. Additionally, all emerging themes were discussed with other members of the research team, including members of the original InfoRehab study team.

We received ethics clearance from The University of Waterloo Research Ethics Committee, Western University Research Ethics Board, and University of British Columbia Research Ethics Board for the original data collection and analysis. Ethics clearance was received from the University of Waterloo Research Ethics Committee for the subsequent focus group and analysis.

## Results

Each patient included in the analysis experienced between one and three transitions during their care journey (***Table 1***). Patients and their caregivers were interviewed at each transition point, where possible. Family caregivers were children (n = 7), the patient’s spouse (n = 5), or children-in-law (n = 2). In some cases, interviews were completed with more than one caregiver per patient. Health care providers consisted of case managers (n = 10), registered nurses (n = 8), medical doctors (n = 3), physiotherapists (n = 13), occupational therapists (n = 9), practical nurses (n = 6), clinical nurse leaders (n = 3), surgeons (n = 2), administrative staff (n = 3) and physiotherapist assistants (n = 2). Most often, multiple providers were interviewed for each patient at each transition point along the care journey.

**Table 1:** Patient Transitions.


PATIENT	SITE	LOCATION PRIOR TO FRACTURE	TRANSITION

Patient 1	Mid-Sized Urban	Home	Acute Care ➔ Inpatient Rehabilitation ➔ Retirement Home ➔ Home Care

Patient 2	Mid-Sized Urban	Home	Acute Care ➔ Inpatient Rehabilitation ➔ Home Care

Patient 3	Mid-Sized Urban	Home	Acute Care ➔ Home Care

Patient 4	Mid-Sized Urban	Home	Acute Care ➔ Home Care

Patient 5	Mid-Sized Urban	Home	Acute Care ➔ Inpatient Rehabilitation

Patient 6	Mid-Sized Urban	Long Term Care	Acute Care ➔ Long Term Care

Patient 7	Rural	Home	Acute Care (Rural) ➔ Retirement Home ➔ Home (no home care)

Patient 8	Rural	Home	Acute Care (Rural)➔Long Term Care ➔ Home (no home care)

Patient 9	Rural	Home	Acute Care (Urban) ➔ Acute Care (Rural) ➔ Home Care ➔ Out Patient Rehab

Patient 10	Rural	Home	Acute Care (Rural) ➔ Home Care

Patient 11	Rural	Home	Acute Care ➔ Acute Care (Rural) ➔ Long Term Care

Patient 12	Rural	Retirement Home	Acute Care (Rural) ➔ Acute Care (Urban) ➔ Acute Care (Rural) ➔ Retirement Home (with home care)

Patient 13	Rural	Home	Acute Care (Rural) ➔ Long Term Care

Patient 14	Rural	Home	Acute Care (Rural) ➔ Home Care ➔ Out Patient Rehab

Patient 15	Rural	Home	Acute Care (Rural) ➔ Home Care ➔ Out Patient Rehab

Patient 16	Large Urban	Home	Acute Care ➔ Home Care

Patient 17	Large Urban	Home	Acute Care ➔ Inpatient Rehab ➔ Assisted Living ➔ Home Care

Patient 18	Large Urban	Home	Sub-Acute Care ➔ Readmission ➔ Inpatient Rehab ➔ Home Care

Patient 19	Large Urban	Home	Acute Care ➔ Inpatient Rehab ➔ Home (no home care)


After completion of the secondary analysis, a focus group interview was conducted with a group of 15 participants involved in health system-level initiatives related to the care of older adults. The participants spoke to the care of older adults in the same mid-sized urban location originally investigated in the InfoRehab study. The participants were health care providers or managers and worked in Home and Community Care (n = 2), Regional Community Services (n = 1), Community Support Services (n = 1), Primary Care (n = 1), Specialized Geriatric Services (n = 4), the Alzheimer’s Society (n = 1), Regional Outreach Programs (n = 1), Long Term Care (n = 2), Hospital Clinical Services (n = 1) and the Local Health Integration Network (regional health authority in Ontario, Canada; n = 1). The focus group interview participants were provided with an overview of the secondary analysis findings, accompanied by a verbal description of the results. Participants were asked to comment on how the results matched their experiences in the current health care systems, and about gaps within the findings.

Seven key themes relating to transitional care experiences were identified in the secondary analysis. These themes are listed in ***Table 2***, below.

**Table 2:** Emerging Themes.


	THEME

1	Multiple different providers contributed to patient and family caregiver confusion

2	Family caregivers were not considered an important partner in the patient’s care

3	System-related issues impacted care experience

4	Patients and family caregivers felt uninformed

5	Transitions increased stress in patients and family caregivers

6	Care was not tailored to patient needs

7	Providers faced barriers in getting adequate information


These themes were discussed by the focus group participants, who ultimately concluded that the results were not surprising.

The analysis revealed that certain themes appeared to be more relevant at select transition points in a patient’s care. Similarities seemed to exist in transitional experiences *to home settings* (acute care to home, and inpatient rehabilitation to home), and in transitional experiences *to other formal care settings* (acute care to inpatient rehabilitation, and acute care to long term care). The results of the analysis therefore, will be summarized within the categories of ‘Transitions to Formal Care Settings’ and ‘Transitions to Home’. Importantly, many of the themes were relevant across all transitional care settings, however, they were found to be more pertinent within particular transitions and as a result are presented in the two categories.

### Transitions to Formal Care Settings

In transitions to formal care settings, such as long-term care and inpatient rehabilitation, similarities in experience emerged through the secondary data analysis. These similarities were largely related to the first three emerging themes (***Table 2***). Overall, patients, caregivers and health care providers seemed most concerned with their lack of understanding about the patient’s care and transition process in transitions to other formal care settings.

#### Multiple different providers contributed to patient and family caregiver confusion

In transitions to and from formal care settings, patients and caregivers were especially troubled by confusion regarding the roles of the multiple providers involved in their care, and commonly described the difficulty they experienced in differentiating between various types of staff or providers in acute care and inpatient rehabilitation settings:

“It’s not that I don’t remember, I wouldn’t know anyway because you don’t know whether they’re a nurse, health provider or whether they’re just one of the people that serve the meals. You don’t know, because there’s no indication on their uniform…” (Mid Urban, Patient 0, Acute to Inpatient Rehabilitation)

One health care provider also identified this issue, explaining that adding more providers may not be the best solution for older people:

“There’s so many people already involved with them and because there’s seniors you go in and say ‘did your physiotherapist…?’, ‘well which one’s she?’ So they see an OT, they see a PT, they see their staff nurse, they see the unit resource nurse, they see the doctor, and they see me… and they’re totally confused.” (Mid Urban, Case Manager 4, Acute Care)

Multiple providers raised the issue of fragmentation of care. Additionally, patients and caregivers explained that the lack of provider communication meant that no one had a view of the whole picture, resulting in greater confusion about their care and transition process. Further compounding this problem with frustration over the inconvenience of having to repeat their story to each provider as they transition to new care settings. A focus group participant expanded upon this idea, and wondered about how consistency in providers impacts transitional care:

“I think it would be interesting to look at the [Rural Hospitals] Model, where the doctor is the doctor. Like he’s in and out of the hospital… I just think there’s a disconnect. Like you just see it, it’s like coming into a whole brand new setting, nobody knows you, and when you observe – and I’m just observing here—[Rural locations] where there is no hospitalist, the family doctor goes in, knows you, knows what that plan is, knows when the next appointment is, knows when you’re going to be discharged, knows what you’re going to do. And I have to say, I do wonder what hospitalists have done to the whole impact of transition of care…” (Focus Group Participant 8)

#### Family caregivers were not considered an important partner in the patient’s care

Throughout all transitions of care, caregivers commented on how little they were considered in the patient’s care. This was especially prominent in transitions to formal care settings. Caregivers frequently felt as though they were not included as a part of the patient’s care, and their needs were not addressed by the system:

“you know, what seems to get lost in it, is somebody caring for an elderly person with a bad hip, you know, it just seems to be at the bottom of the totem pole.” (Rural, Caregiver I202, Acute to Long Term Care)

One health care provider explained that the health care system asks caregivers to fit themselves into the system’s rules and schedules, rather than working with the caregiver to find a mutually agreeable solution:

“…we’re asking them as family to leave behind all of their day to day routines and jump into our culture, our community, and follow our rules. ‘The patient has to be out of here by eleven o’clock.’ ‘But I work?’ ‘Well you have to take a day off.’…” (Mid Urban, Case Manager 7, Inpatient Rehabilitation)

Additionally, in inpatient rehabilitation and acute care settings, providers rarely interacted with caregivers. Providers explained that since most caregivers visit after regular working hours, they never get the chance to talk in person.

#### System-related issues impacted care experience

Across all transitions, patients and caregivers felt that system issues negatively impacted their quality of care. Many patients and caregivers in transitions to formal settings, such as acute care and inpatient rehabilitation, felt that nurses were too busy to provide adequate and personalized care, or answer their questions in detail:

“I can see how people fall through the cracks for their condition. If it isn’t highlighted there’s some part of it slips by and it’s not covered, and then it doesn’t get covered the next day, and eventually it might become an issue but by then they are two days late.” (Mid Urban, Patient 3, Acute to Home)

Patients explained that many providers in these settings were unfamiliar with their unique needs, conditions, and personality, making it difficult to get the care they need, and ask questions about their care. The impersonal and rushed care that patients reported experiencing in the acute care and inpatient rehabilitation settings left the patient feeling doubtful about their care and recovery status:

“They’re getting too far away from the people that they’re supposed to be taking care of without them even being aware of it themselves. They don’t come and take the time to listen to you… and then they say, ‘any questions?’ But by that time your head’s so full of information from them, how can you sort it out to ask a question?.” (Large Urban, Patient 4, Inpatient Rehabilitation to Home)

Patients and caregivers who had previous experience in the health care system explained that this experience made it slightly easier to navigate the system, especially in acute and inpatient rehabilitation settings, where acquiring information was easier with knowledge of where to look and who to ask. One health care provider commented on why the health system complicates transitional care for patients, caregivers, and providers:

“…it’s a system that you don’t know anything about until you need to be involved in it or you’ve had another family member be involved in it. And the second thing is, it’s not a system. A system is made up of parts that interact and in the health care environment they don’t interact. You know, when you’re in hospital there’s no communication with the people outside.” (Mid Urban, Case Manager 1, Acute Care)

### Transitions to Home

Commonalities also existed between transitions to home, whether from acute care or inpatient rehabilitation settings, specifically in relation to themes four through seven (***Table 2***). In transitions to home, patients, caregivers, and health care providers generally focused on their feelings of being unprepared.

#### Patients and family caregivers felt uninformed

In all transitions, information about the patient’s condition, care, and transition trajectory was not readily available or provided to patients or caregivers. This lack of information was especially relevant for patients and caregivers transitioning from acute care to home, or inpatient rehabilitation to home. Caregivers who experienced transitions from acute care explained that they were often not given any information. The patient however, was often overloaded with information while sedated, tired, or otherwise preoccupied. On occasion, patients were provided with pamphlets or fact sheets containing general information about hip fracture and surgery, but were left to interpret this information independently. In some cases, these pamphlets and fact sheets were even passed along to them by patients who had received other procedures, such as hip replacements. Many of the questions patients had however, pertained to their *unique* care needs and recovery process. Therefore, the standard written information provided by pamphlets was not helpful in answering patients’ questions or preparing them for returning to home.

The difficulty that patients and caregivers faced in getting information from providers contributed to a sense of being instructed through their care, rather than being actively involved in their care decisions. Patients and caregivers in transitions from acute care or inpatient rehabilitation to home did not feel involved in the decisions made about their care.

“I guess I could have been more involved but I just kind of got the impression the decisions were already made… I mean I asked a lot of questions and whatever but I suppose if I was really dead set against them I could have made a fuss but I didn’t really feel that it was really up to me.” (Mid Urban, Caregiver 0, Inpatient Rehabilitation to Home)

Patients and caregivers often attributed this lack of involvement to the speed at which transition decisions needed to be made. They explained that the lack of involvement in their care and the speed at which their transition occurred left them feeling unprepared for their return to home. One focus group participant commented on the uncertainty faced by patients and caregivers returning home, explaining that it is understandable to feel that way given the circumstances:

“What is also interesting to me, when I think about all of the tools that we have access to as health care professionals to coordinate care, and we often don’t even… So if I don’t feel confident, imagine the caregiver’s going home… Because it is about bolstering confidence and making them feel supported right?” (Focus Group Participant 6)

#### Transitions increased stress levels in patients and family caregivers

During transitions, caregivers felt overwhelmed with their increased responsibilities. This was especially relevant for transitions to home. Caregivers of patients who were moving home were often left to prepare the environment for the patient’s return, ensure that the patient was properly cared for, and arrange all follow-up appointments. Caregivers explained that this was a difficult task, given that they had little information on what the patient might need.

Patients and caregivers transitioning from acute care settings seemed most concerned with the lack of involvement in their care transitions. In this transition, patients and caregivers wished they had more tailored information about the process and future care:

“I would like to know how… he’s progressing approximately. Will he be out with, say, a week if he really works hard? Is there any other place that he could go to for rehabilitation besides here? Do they have other hospitals that have rehab?” (Large Urban, Caregiver 2, Acute to Inpatient Rehabilitation)

In transitions from the acute setting, it was especially important for patients and caregivers to understand their specific and unique care trajectory. In contrast, patients transitioning from inpatient rehabilitation seemed more concerned about the lack of constant professional support they might experience at home, which resulted in uncertainty regarding their ability to succeed at home:

“But there’s a big “but” in there that, you know, will I able to manage all right.” (Large Urban, Patient 3, Inpatient Rehabilitation to Home)

Patients and caregivers transitioning from inpatient rehabilitation were often apprehensive about transitioning from an environment with high support, to one of low support. Patients worried that they may not be ready to return home or may not be successful in recovering at home.

#### Care was not tailored to patient needs

Many patients transitioning from acute care to home, or inpatient rehabilitation to home, commented on the fact that the care they received in acute care and inpatient rehabilitation settings was not helpful within their next setting, especially when their next setting was home. Patients explained that these uncertainties left them feeling anxious and unprepared for the transition:

“Always in the back of your mind you’re wondering, like, what are my limits? And nobody really has an answer for that because I guess it depends on your particular hip problem. So you have to judge that for yourself. And hopefully you’ll do the right thing.” (Large Urban, Patient 5, Inpatient Rehabilitation to Home)

However, after returning home, many patients explained that these feelings of uncertainty were reduced by the efforts of the home care providers, who attempted to personalize their therapy to their particular lifestyle and home environment.

#### Providers faced barriers in getting adequate information

Providers in all settings explained that electronic medical records were very useful for retrieving information about the patient. However, without compatible systems across different settings, providers had limited access to a patient’s previous records. To address these gaps, each provider in a new setting completed their own assessment of the patient. In addition to these assessments, providers often relied on patients and caregivers for information that they could not retrieve from a previous setting. This added to the workload of the providers, who already felt constrained by the amount of time that they could afford to spend with patients. The lack of communication also added stress to the patient, who continually had to reiterate information to each new provider, while still feeling that no one had a strong grasp of their condition or recovery needs.

This gap in information sharing was particularly problematic for home care providers. Providers in the home explained that they often saw a patient for an initial assessment without any previous knowledge of their condition.

“So I know that discharge planning can change quite quickly… Ideally, yes, we like to know all those - that bit of information, that everything was in place prior to the client coming home. But I know it’s not done and I know it’s because you’ve got the O.T. doing her thing, the physio doing her thing in the community… I mean, there’s just so many pieces happening, that supposedly somebody’s looking at the big picture.” (Large Urban, Case Manager 4, Acute to Home)

Home care providers explained that not having access to a patient’s charts resulted in a longer intake assessment, which ultimately limits the time that they can spend with the patient working on recovery treatments and strategies. Providers working in the home setting went on to explain that without a common chart, the patient is assessed and treated in pieces, which prevents providers from understanding the patient as a whole. A focus group participant explained that communication across settings is still a significant challenge in the health care system:

“There’s still some challenges with acute care seeing that long term care is in the circle of care when you send somebody to the hospital… because we have a responsibility to ensure that our staff are able to talk to the hospital staff…. And really it’s just good care, it’s in the best interest of the client, of the resident, of the patient. That’s really why we want to have that conversation, it’s not we’re trying to be nosey about somebody, it’s just that we really need to know.” (Focus Group Participant 3)

Quotes such as the one above point to the importance of mechanisms for collaboration and coordination between care providers, both with and across care settings.

## Discussion

This analysis demonstrated that transitional care experiences differed by transition type, these experiences were categorized into transitions to other formal care settings (i.e., long term care, inpatient rehabilitation) and transitions to home. The results of the focus group interview suggested that many of the themes from the secondary analysis may best be addressed through enhancing consistency and coordination of care across settings.

### Lack of Coordination and Communication in Transitions to Formal Care Settings

Being uninformed and uninvolved was a common theme in transitions to other formal care settings, such as long-term care and inpatient rehabilitation, leaving patients and caregivers confused about their transition, their care, and their rehabilitation. Previous InfoRehab studies have reported on the difficulty that patients, family caregivers, and health care providers have in obtaining the information they need [19,20,26,27,28,29,30]. The focus group participants suggested that this sense of confusion may be related to the number of providers involved in these transitions. McLeod and colleagues [19] reported that the use of multidisciplinary teams is a common strategy intended to enhance the care of complex patients, but noted that as the patient’s complexity grows, the size of their ‘circle of care’ also grows. The findings of this analysis indicated that while health care providers in inpatient rehabilitation settings found the multidisciplinary approach beneficial, patients and caregivers still seemed to find the variety of providers confusing. Further, patients and family caregivers in both acute care and inpatient rehabilitation settings worried that, in segmenting the patient’s care to various different specialized professionals, no one was seeing the whole picture. As Toscan and colleagues [26] explained, health care providers involved in the InfoRehab study seemed to feel less responsible for the patient’s care as the size of their circle of care grew. The feelings of being uninformed and uninvolved were consistent throughout the experiences of patients, caregivers, and health care providers transitioning to other formal care settings.

### Feeling Unprepared for Transitions to Home Settings

The secondary analysis demonstrated that patients and caregivers transitioning from acute or inpatient rehabilitation to home often felt uncertain about whether the ‘cookie cutter’ care they received would help them to adjust in their unique home setting. Toscan and colleagues [20] explained that in transitions to home, the biggest challenge is isolation and doubt in one’s own abilities, suggesting that this uncertainty can be a significant hurdle for patients and caregivers transitioning to home settings. Transitions to home were particularly stressful for family caregivers, who were suddenly responsible for a majority of the patient’s care. Caregivers were expected to manage the transition to home, which involved a variety of care tasks. These expectations often came without any flexibility or consideration of the caregiver’s availability and without direct instruction about how best to provide the patient with the care they need at home. Toscan and colleagues [26] explain that this reliance on family caregivers results in unease and stress, especially since most caregivers lack the skills and knowledge to adequately care for the patient at home. In transitions to home settings, patients, caregivers, and health care providers seemed to feel very unprepared and uncertain about the care of the patient in the future. This feeling of being unprepared was especially hard for the caregivers, who felt very unsure about how to care for the patient at home. Weaver, Perloff & Walters [31] concluded that, in transitions to home, caregiver stress is associated with a lack of information.

## Applications for an Integrated Care Approach to Transitional Care

Integrated care approaches have been suggested as promising solution to enhance coordination and experiences across transitions of care [13–14]. It has been suggested that integration in the health care system is too complex for a ‘one-size-fits-all’ solution [32]. Numerous contextual factors influence health care delivery, and these factors vary by population, setting, and a series of other factors [30]. Integration of care across different health settings should consider the various factors that uniquely influence that setting. In other words, solutions focused on integrating care for people transitioning to formal care settings should consider the confusion experienced by patients and their caregivers, while solutions for transitions to home should consider the patients’ and caregivers’ feelings of being unprepared. While solutions should consider unique and individual factors, there are some broader characteristics that can be included to support integrated approaches, including person-centeredness and information sharing [32]. Furthermore, the results from this study provide important findings for practice change including improving discharge planning practices, enhancing information sharing between health care organizations, clarifying roles among health care providers, and providing adequate education to and engaging patients and their caregivers [22].

## Strengths and Limitations

A potential limitation of this work is that only one focus group interview was conducted with a group of participants representing only one of the geographic locations in which the original InfoRehab study had been conducted. However, the focus group allowed for inclusion of a range of individuals involved in system solutions, and with backgrounds in a variety of health care settings and professions. One strength of this research is the amount of data analyzed. Another strength would be that the interviews were conducted with participants within 72 hours of the transition. Interviews were completed by research assistants at each study location. Each research assistant used the same interview guide and received appropriate training on qualitative interviewing. Another potential limitation is that a single researcher completed the analysis. To mitigate this limitation, the researcher (LB) examined the emerging themes in relation to original InfoRehab project findings and discussed all emerging themes with InfoRehab research team members.

## Conclusions

The findings of this research suggest that patients, caregivers, and health care providers experience transitions between various settings of care differently, suggesting the need for tailored approaches to improving care transitions and system integration. Older adults with complex conditions, including hip fractures, receive care from multiple providers and experience multiple care transitions. Future research should further explore the ways in which other transition settings may impact patient, caregiver, and health care provider experience, and quality of care, for a variety of complex health conditions.
